# Fatty acid oxidation is associated with proliferation and prognosis in breast and other cancers

**DOI:** 10.1186/s12885-018-4626-9

**Published:** 2018-08-09

**Authors:** Aziz Aiderus, Michael A. Black, Anita K. Dunbier

**Affiliations:** Centre for Translational Cancer Research and Department of Biochemistry, University of Otago, Dunedin, 9054 New Zealand

**Keywords:** Fatty acid oxidation, Gene signature, Cancer, Prognosis, Tumour-normal, CPT1A, Proliferation, Migration

## Abstract

**Background:**

Altered cellular metabolism is a hallmark of cancer but the association between utilisation of particular metabolic pathways in tumours and patient outcome is poorly understood. We sought to investigate the association between fatty acid metabolism and outcome in breast and other cancers.

**Methods:**

Cox regression analysis and Gene Set Enrichment Analysis (GSEA) of a gene expression dataset from primary breast tumours with well annotated clinical and survival information was used to identify genesets associated with outcome. A geneset representing fatty acid oxidation (FAO) was then examined in other datasets. A doxycycline-inducible breast cancer cell line model overexpressing the rate-limiting enzyme in FAO, carnitine palmitoyl transferase 1A (*CPT1A*) was generated and analysed to confirm the association between FAO and cancer-associated characteristics in vitro.

**Results:**

We identified a gene expression signature composed of 19 genes associated with fatty acid oxidation (FAO) that was significantly associated with patient outcome. We validated this observation in eight independent breast cancer datasets, and also observed the FAO signature to be prognostic in other cancer types. Furthermore, the FAO signature expression was significantly downregulated in tumours, compared to normal tissues from a variety of anatomic origins. In breast cancer, the expression of *CPT1A* was higher in oestrogen receptor (ER)-positive, compared to ER-negative tumours and cell lines. Importantly, overexpression of CPT1A significantly decreased the proliferation and wound healing migration rates of MDA-MB231 breast cancer cells, compared to basal expression control.

**Conclusions:**

Our findings suggest that FAO is downregulated in multiple tumour types, and activation of this pathway may lower cancer cell proliferation, and is associated with improved outcomes in some cancers.

**Electronic supplementary material:**

The online version of this article (10.1186/s12885-018-4626-9) contains supplementary material, which is available to authorized users.

## Background

Improved understanding of the molecular features associated with prognosis in primary tumours is key to better management of the disease. High throughput gene expression technologies have facilitated the molecular profiling of tumours and generation of prognostic gene signatures [1]. However, particularly in breast cancer, many of these gene signatures contain genes that are strongly correlated with proliferation, and the biology underlying their enrichment is poorly understood [2, 3].

Alterations in cellular metabolism and energetics are hallmarks of cancer [4, 5]. One of the earliest observations of altered tumour metabolism was increased aerobic glycolytic flux, termed the Warburg effect [6]. A large number of studies focusing on this pathway have subsequently found that glycolysis serves energetic and anabolic roles for cell division [7]. Technological advances such as metabolomics and isotope tracing have been employed to study cellular metabolism and have revealed that other metabolic pathways that are co-opted by tumours to support cancer cell division [8]. For example, some tumours have increased reliance on the oxidation of the amino acid glutamine [8], however, the role of fatty acid metabolism in cancer remains unclear. Specifically, while the role of fatty acid synthesis is better understood, how FAO affects tumour biology remain contentious [9]. Furthermore, even though most of these metabolic pathways have been extensively studied using in vitro and in vivo systems, their association with patient outcome remains to be determined.

In this study, we report the generation and validation of a gene signature involved in fatty acid oxidation (FAO) and prognosis in breast, and several other cancer types. Our findings suggest that pharmacologic agents that upregulate FAO may have therapeutic potential.

## Methods

*Cox regression analysis and gene set enrichment analysis* – Gene expression data and associated clinical information from the METABRIC study [10] was obtained through Sage Bionetworks with appropriate ethical approval (University of Otago Human Ethics Approval H16/092) and was used as the training dataset. All data analysis was performed using the R Software [11]. Only patients with ER-positive tumours that received radiation and/or endocrine therapy (*n* = 973) were included. Gene expression data was collapsed so that each gene was represented by a single probe using the *collapseRows* function from the ‘WGCNA’ package [12]. Cox regression analysis was performed using the *coxph* function available from the ‘survival’ package [13]. The *p* values associated with the hazard ratios for each gene were adjusted for multiple comparisons by the false discovery rate (FDR) method [14]. Genes and associated *p* values were then sorted in ascending order (most-to-least significant) and pre-ranked gene set enrichment analysis [15] was performed using the KEGG database [16]. Hierarchical clustering and heatmaps were generated using the *heatmap.2* function with Euclidean as the distance metric and complete linkage as the linkage criterion.

*Survival, multivariable Cox regression, and logistic regression analyses* – All survival analyses were performed in RStudio using the ‘survival’ package, or using the KMplotter online software [13, 17]. Statistical significance for differences between survival curves was calculated using the log-rank test [13]. Multivariable Cox regression analysis was conducted using available clinico-pathologic factors, depending on the datasets analysed. For survival analysis, the average expression of the 19-gene fatty acid oxidation signature was calculated for each patient, and stratified into two groups - above or below the median. For validation analysis on independent breast cancer datasets, the log-rank *p* values were adjusted for multiple comparisons using the FDR method. To estimate the odds-ratio of achieving pathologic complete response to neoadjuvant chemotherapy based on low (below median) or high (above median) expression of the fatty acid oxidation signature expression, logistic regression was performed. The final meta-analysis odds ratio was obtained by taking the average value of the point estimates and confidence intervals. The datasets used for the validation analysis of the fatty acid oxidation signature, conducting logistic regression on neoadjuvant chemotherapy breast cancer trials, and tumour-normal analysis are summarised in Additional file 1: Table S1.

In silico *CPT1A expression analysis in breast tumours and cell lines* – Datasets used for validation analysis of the FAO signature were also used to investigate the expression of *CPT1A* in breast tumours. For breast cancer cell lines, two datasets were analysed for expression of *CPT1A*: (i) quantile normalised with gene level summary data [18] from E-MTAB-181 [19] was accessed from ArrayExpress, and (ii) GSE57083 accessed from NCBI GEO where the RMA-normalised [20] expression matrix was used to calculate the average expression of *CPT1A* for each cell line based on the values from four probesets: 203633_at, 203634_s_at, 210687_at and 210688_s_at on the Affymetrix Human Genome U133 Plus 2.0 array.

*CPT1A overexpression in MDA-MB231 cell line –* The coding sequence for *CPT1A* (NM_001876.3) was accessed from the NCBI Nucleotide portal and primers were designed to amplify the entire sequence. Total RNA from MCF10A normal mammary epithelial cells were converted to cDNA, and high-fidelity PCR performed to amplify the *CPT1A* coding sequence. PCR products were gel-purified, digested with *Sac*II and *Xba*I, and ligated into a doxycycline-inducible plasmid downstream of a Tet-response element (TRE). The *CPT1A* coding sequence was Sanger sequenced to verify that no mutations were introduced during the cloning procedure.

*Doxycycline dose response and time course characterisation* – For dose response and time course analysis of doxycycline (Dox)-induced CPT1A expression, selected MDA-MB231 pTRE-CPT1A clones were seeded in 6 well plates and induced with 2 μg/mL Dox for 48 h. Whole cell lysates were prepared and resolved by SDS-PAGE. Proteins were transferred to a PVDF membrane, blocked for one hour with 5% (*w*/*v*) milk, and incubated with a CPT1A antibody (Abcam 128,568, mouse anti-human, 1 μg/mL) overnight. Membranes were washed three times with 1 X TBST solution for 10 mins per wash, and then incubated with secondary antibody (Amersham NA931V, sheep anti-mouse 1:10,000) for one hour at room temperature. Membranes were washed as described above, and incubated with 2 mL of enhanced chemiluminescent solution for 5 mins, prior to imaging on the Odyssey LiCor system.

*Proliferation rate analysis between basal and CPT1A overexpression in MDA-MB231 cell line* – To investigate whether *CPT1A* overexpression affects the proliferation rate of MDA-MB231 cells, TetOn parental and pTRE-CPT1A clones were seeded in 6 well plates and induced with 2 μg/mL Dox for 48 h. Cells were then seeded in 96 well plates (1000 cells/well) with and without Dox, and real-time proliferation was monitored using the IncuCyte live cell imaging system (Essen Bioscience).

*Wound healing rate analysis between basal and CPT1A overexpression in MDA-MB231 cell line* – To compare the wound healing migration rates between basal and CPT1A overexpression in MDA-MB231 cells, TetOn parental and pTRE-CPT1A clones were seeded in 6 well plates and induced with Dox for 5 days. Cells were then seeded in an Essen ImageLock 96-well plate at full confluency (60,000 cells/well). The following day, a scratch was created through the middle of each well using the Essen WoundMaker and fresh media was replaced in each well. Real time wound healing migration rates were monitored using the IncuCyte live cell imaging system.

*Soft agar clonogenic growth analysis between basal and CPT1A overexpression in MDA-MB231 cell line* – To investigate whether CPT1A overexpression affects anchorage-independent colony formation in MDA-MB231 cells, TetOn parental and pTRE-CPT1A clones were seeded in 0.3% agar and layered over 0.6% agar. The soft agar assay was conducted for two weeks, with media changes every 2–3 days. Cells were then fixed with 10% methanol, stained with 0.1% crystal violet and the number of colonies counted under a phase contrast microscope.

## Results

*Cox regression and gene set enrichment analysis to identify genes associated with breast cancer disease-specific survival* – To identify genes and pathways that are significantly associated with disease-specific survival in the METABRIC cohort, we performed Cox regression analysis on gene expression data from 973 primary breast tumours from the METABRIC study. The resulting *p-*values were adjusted using the false discovery rate (FDR) method. The genes were then sorted according to their adjusted *p*-values, and pre-ranked GSEA analysis using the KEGG database was performed.

Table 1 summarises the KEGG pathways that were significantly enriched in the Cox regression analysis. Several of the enriched pathways such as ‘DNA replication’, ‘Pyrimidine Metabolism’ and ‘Base Excision Repair’ contain genes correlated with proliferation, which has been shown to be highly prognostic in ER-positive breast cancer [21, 22].

**Table 1:** KEGG pathways associated with disease-specific survival in METABRIC training cohort

KEGG pathways	Enrichment score	Normalised enrichment score	Nominal *p*-value	FDR q value
Valine, leucine, isoleucine degradation	0.53	1.74	0	0.03
**Base excision repair**	0.55	1.71	0	0.03
**Pyrimidine metabolism**	0.48	1.68	0	0.03
**DNA replication**	0.52	1.66	0	0.03
Glyoxylate and dicarboxylate metabolism	0.58	1.63	0.002	0.03
**Oocyte meiosis**	0.46	1.62	0	0.03
Glycine, serine, threonine metabolism	0.51	1.61	0.002	0.03
Proteasome	0.48	1.58	0.002	0.04
*Fatty acid metabolism*	0.48	1.58	0.002	0.04

Proliferation associated pathways are in bold, while the Fatty Acid Metabolism pathway analysed further in this study is in italic

**Fig. 1:**
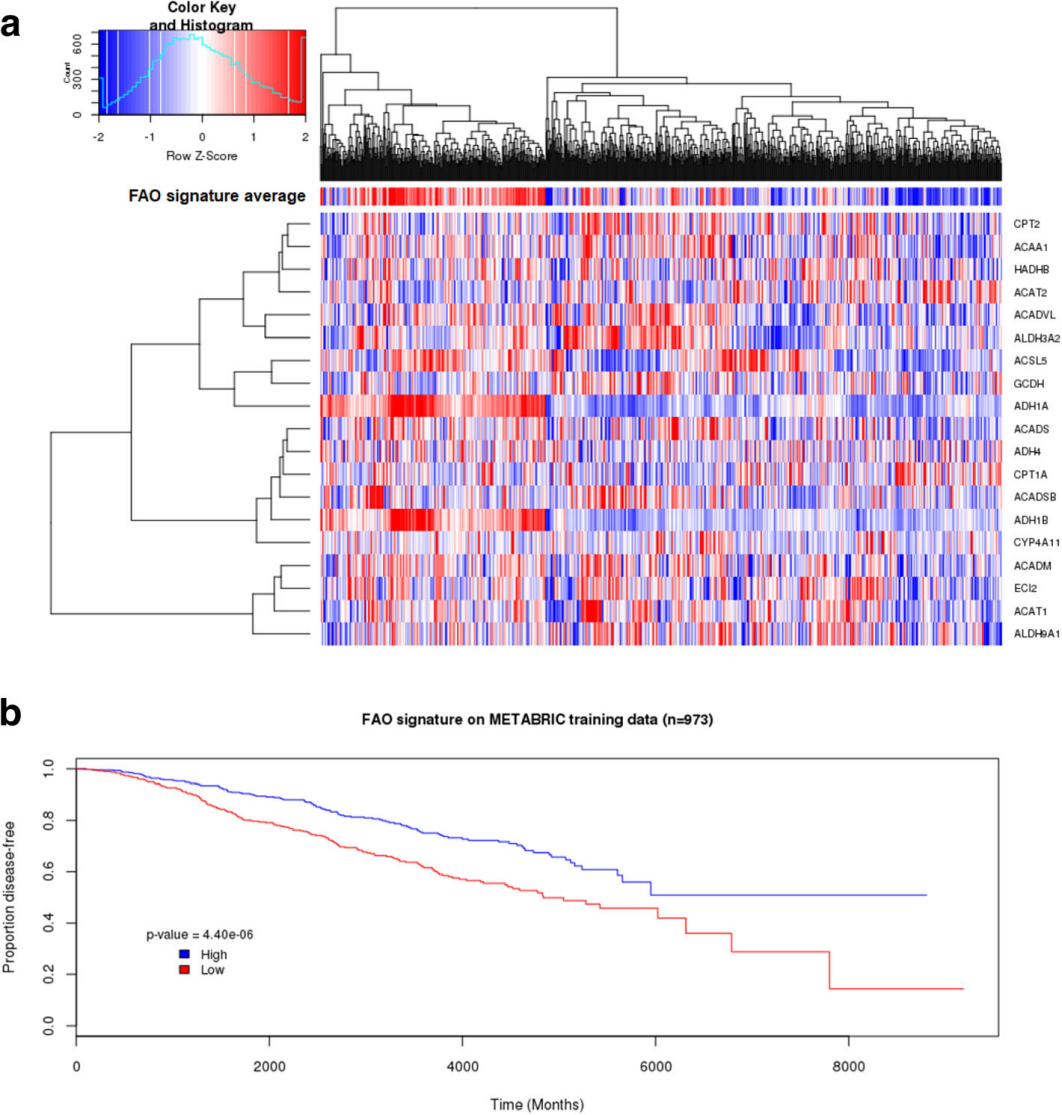
Expression of the 19-gene FAO signature is prognostic in the METABRIC training cohort**. a** Heatmap depicting expression of the FAO signature in the METABRIC training cohort. Rows correspond to the expression of indicated genes, while columns correspond to each patient (*n* = 973). Red and blue correspond to low and high gene expression respectively, on a continuous scale.**b** Kaplan-Meier survival curve of FAO signature expression in the METABRIC training cohort. Outcomes in patients with high (blue) expression of the FAO signature differs significantly than patients in the low (red) group (log-rank *p* = 4.40e-06). High and low expression was defined as patients with average FAO signature expression value above and below the median cutoff, respectively

For further investigation we focused on the KEGG Fatty Acid Metabolism pathway, which was one of the gene sets that was enriched (nominal *p* = 0.002, FDR adjusted *q* = 0.03) (Fig. 1a). Of note, 19 out of 42 genes in this gene set were defined as core-enriched based on the output of the analysis, which means they were over-represented at the top of the pre-ranked gene list provided and contributed the most to the enrichment of this pathway. These 19 genes are referred to as the “fatty acid oxidation (FAO)” signature hereafter (Table 2). Included in this signature were genes which have previously been identified as the core components of the fatty acid beta-oxidation pathway, such as *CPT1A*, *CPT2*, *ACADM*, *ACADSB,* and *ACADVL* [23].

**Table 2:** Gene symbols and names for members of the 19-gene FAO signature

Gene symbol	Gene name
ACAA1	acetyl CoA acyltransferase 1
CPT1A	carnitine palmitoyl transferase 1A
ACADM	acyl CoA dehydrogenase, C-4 to C-12 straight chain
GCDH	glutaryl-CoA dehydrogenase
ACADS	acyl CoA dehydrogenase, C-2 to C-3 straight chain
ACAT2	acetyl CoA acetyltransferase 2
ECI2	enoyl CoA isomerase 2
ACAT1	acetyl CoA acetyltransferase 1
ACADSB	acyl CoA dehydrogenase, short/branched chain
CYP4A11	cytochrome P450 family 4 subfamily A member 11
ACADVL	acyl CoA dehydrogenase, very long
ADH1A	alcohol dehydrogenase 1A, (class I), alpha polypeptide
CPT2	carnitine palmitoyl transferase 2
HADHB	hydroxyacyl CoA dehydrogenase/3-ketoacyl CoA thiolase/enoyl CoA hydratase, trifunctional protein beta subunit
ADH1B	alcohol dehydrogenase 1B, (class I), beta polypeptide
ALDH9A1	alcohol dehydrogenase 9, family member A1
ACSL5	acyl-CoA synthetase long-chain family member 5
ADH4	alcohol dehydrogenase 4 (class II), pi polypeptide
ADLH3A2	aldehyde dehydrogenase 3 family member A2

Hierarchical clustering analysis revealed that patients with relatively high or low expression of the FAO signature (denoted as ‘FAO average score’ horizontal bar above the heatmap) were clustered together toward the left or the right of the heatmap, respectively (Fig. 1a). Based on their average FAO signature value, patients in this cohort were stratified into two groups (one made up of patients with expression higher than the median value, and one of patients with expression lower than the median value), and Kaplan-Meier survival curve analysis revealed patients with tumours with high expression (above median cutoff) of the FAO signature had a significantly better outcome (Fig. 1b, log-rank *p* = 4.40e-06). This finding was validated in eight independent datasets, and patients in the low group had univariate hazard ratios between 1.3 and 5.4 for indicated survival metrics (Table 3). These associations were statistically significant in cohorts with distant relapse-free or metastasis-free survival data available. Additionally, the FAO signature expression was also significantly associated with overall survival in two cohorts, and trended towards significance in the TCGA breast cohort (adjusted *p* = 0.097, univariate hazard ratio 1.31 (95% confidence interval 0.95–1.81)). Taken together, these data suggest a robust association between expression of genes involved in FAO and prognosis in breast cancer.

**Table 3:** FAO signature expression is prognostic in independent breast cancer datasets

Dataset	Study	n	Log rank *p* (survival metric)	Hazard ratio (Low vs. High)	Hazard ratio (95% confidence interval)	FDR adjusted *p*
GSE42568	Clarke et al.	104	0.00562 (RFS)	2.24	1.25–4.03	0.007
		104	0.0000958 (OS)	4.05	1.84–8.66	0.000311
GSE20685	Kao et al.	327	0.0118 (DMFS)	1.98	1.26–3.11	0.003
		327	0.00243 (OS)	1.75	1.12–2.27	0.013
GSE46563	Jonsdottir et al.	94	0.000543 (DMFS)	5.46	1.86–16.1	0.002
GSE25066	Hatzis et al.	508	5.98E-06	2.43	1.64–3.62	1.17E-05
TCGA BRCA	TCGA breast cancer	776	0.00387 (RFS)	2.08	1.25–3.47	0.0047
		1096	0.097 (OS)	1.31	0.95–1.81	0.097
GSE22219	Buffa et al.	216	0.00753 (DRFS)	1.82	1.17–2.85	0.0085
BRCA2116	Nagalla et al.	672	6.51E-05 (DRFS)	2.02	1.42–2.88	9.10E-05
GSE21653	Sabatier et al.	266	0.03 (DFS)	1.64	1.06–2.55	0.03

*DFS*, disease-free survival; *DMFS*, distant metastasis-free survival; *DRFS*, distant relapse-free survival; *OS*, overall survival; *RFS*, relapse-free survival

*FAO signature expression is negatively correlated with proliferation* – As low expression of the FAO signature expression is associated with poor outcome in breast cancer patients, and patients with highly proliferative tumours typically have a poor prognosis, we investigated whether the FAO signature expression was correlated with proliferation. Spearman correlation analysis between the FAO and the mitosis kinome score (MKS) [22] - an 11-gene proliferation signature - found a significant, negative correlation between the two signatures in all six cohorts (Spearman’s rho = − 0.27 to − 0.6), suggesting that the FAO signature expression is inversely correlated with tumour proliferation (Table 4).

**Table 4:** FAO signature expression is negatively correlated with proliferation

Dataset	Study	n	Spearman’s rho	*p*
GSE42568	Clarke et al.	104	−0.27	5.17E-03
GSE20685	Kao et al.	327	−0.45	< 3.85E-16
GSE46563	Jonsdottir et al.	94	−0.4	7.70E-15
GSE25066	Hatzis et al.	508	−0.45	< 3.85E-16
GSE22219	Buffa et al.	216	−0.6	< 3.85E-16
TCGA BRCA	TCGA breast cancer	1215	−0.59	< 3.85E-16

Spearman’s correlation analysis between the FAO and MKS proliferation gene signature in breast cancer datasets

**Fig. 2:**
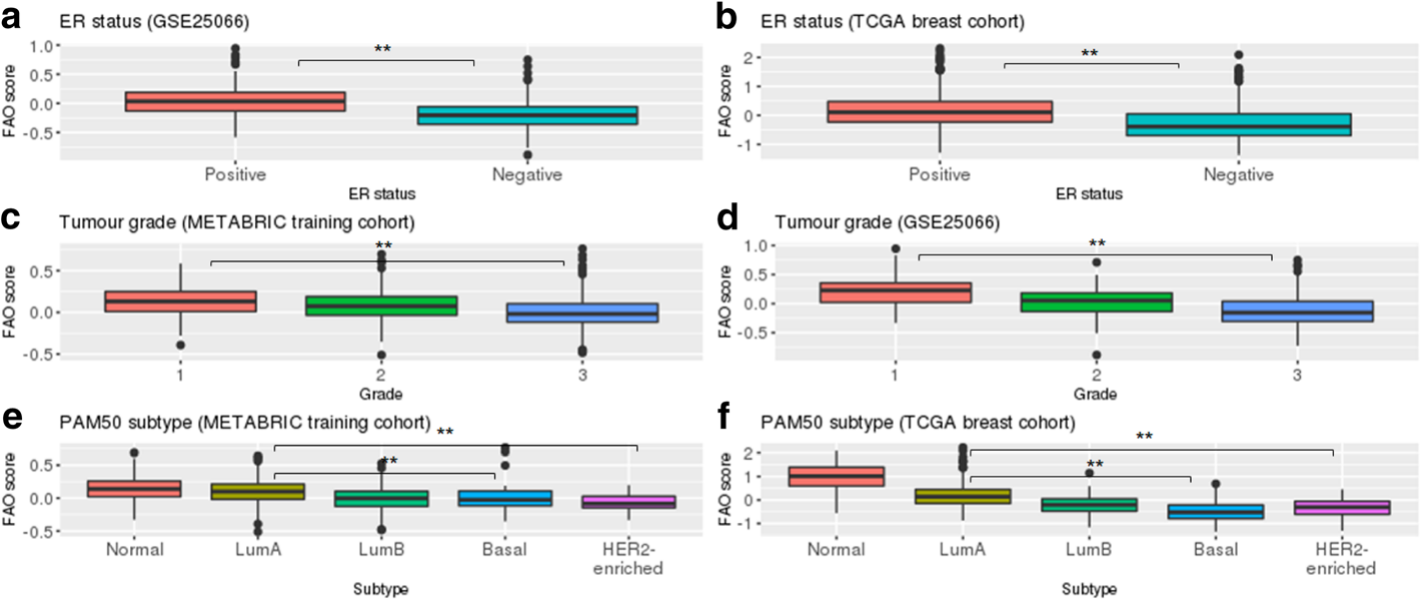
Low FAO signature expression is correlated with clinical features associated with poor prognosis. FAO signature expression is lower in **a**, **b** ER-negative, **c**, **d** higher tumour grade, and **e**, **g** basal-like and HER2-enriched molecular subtypes in different breast cancer datasets. ** Wilcoxon rank sum test *p* < 0.01

*Low FAO signature expression is correlated with clinical features associated with poor prognosis* – We then explored the association between the FAO signature expression and various clinical features. The FAO signature was significantly higher in ER-positive, compared to negative tumours (Figs 2a, b, Wilcoxon rank sum test *p* < 0.01); grade 1 compared to 3 (Figs 2c, d, Wilcoxon rank sum test *p* < 0.01); and luminal compared to basal/HER2-enriched molecular subtypes (Figs 2e, f, g, Wilcoxon rank sum test *p* < 0.01). Hence, the findings suggest that the FAO signature is associated with clinical features that are linked to poor prognosis.

*FAO signature expression is prognostic, independent of standard histopathologic features in breast cancer* – Next, we investigated whether the FAO signature expression provided prognostic information that was independent of standard histopathologic variables. To achieve this, we performed multivariable Cox regression analysis on seven independent breast tumour gene expression datasets.

As summarised in Table 5, after including available established prognostic factors such as tumour grade, size, lymph node and ER status in the Cox model, the FAO signature expression provides significant, independent prognostic information, with patients in the Low group having hazard ratios that range from 1.5 to 5.5, relative to patients in the High group.

**Table 5:** FAO signature expression is prognostic independent of standard histopathological features in breast cancer

Dataset	Study	n^a^	Covariates	Hazard ratio (survival metric)	FDR adjusted *p*
GSE25066	Hatzis et al.	466	Grade, ER status	1.62 (DRFS)	0.0592
BRCA2116	Nagalla et al.	660	Grade, size, lymph node status	1.5 (DRFS)	0.09
GSE42568	Clarke et al.	104	Grade, size, lymph node status	2.55 (RFS)	0.009
GSE22219	Buffa et al.	216	ER status, size, age, lymph node status	1.91 (DRFS)	0.014
GSE46563	Jonsdottir et al.	94	Grade, ER status, size	5.54 (DRFS)	0.009
GSE20685	Kao et al.	327	Age	1.7 (DRFS)	0.009
TCGA BRCA	TCGA breast cancer	776	ER status	1.61 (RFS)	0.09

^a^Of note, the sample sizes in this analysis differ slightly with that presented in Table 3 as there were incomplete clinical information for some patients in certain datasets. *DRFS*, distant relapse-free survival; *RFS*, relapse-free survival

*FAO signature expression is associated with favourable response to short-term, neoadjuvant chemotherapy or aromatase inhibition* – In the previous analyses, several datasets that were used to validate the prognostic performance of the FAO signature included patients who received adjuvant treatment. To determine whether the FAO signature expression is associated with neoadjuvant endocrine or chemotherapy response, logistic regression was performed.

**Fig. 3:**
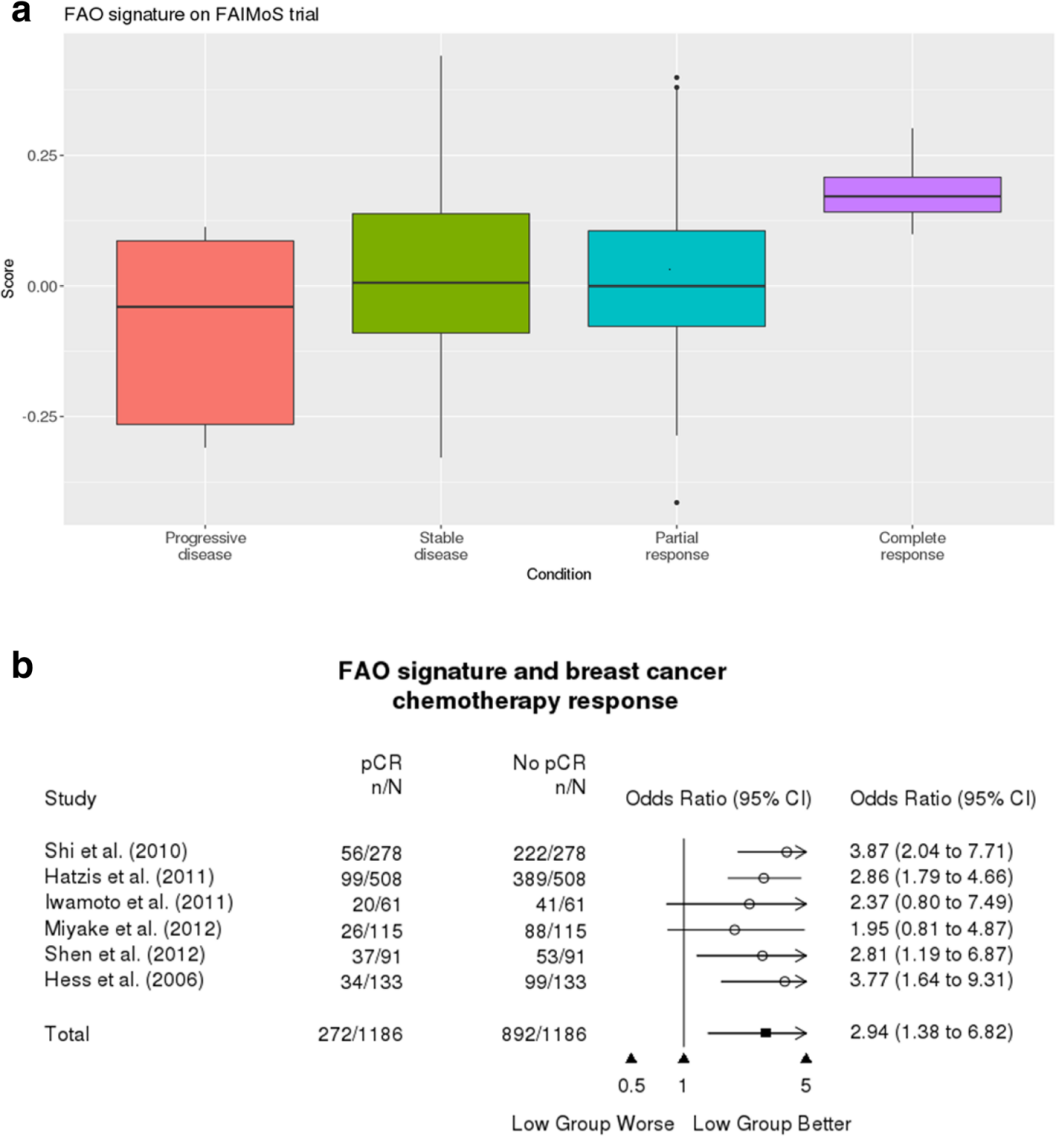
FAO signature expression is associated with response to short-term, neoadjuvant oestrogen deprivation or chemotherapy in breast tumours. **a** High FAO signature expression in pre-treatment primary breast tumours is associated with complete response, compared to progressive disease (Complete response vs Progressive disease Wilcoxon rank sum test *p* = 0.002). **b** Odds ratio of achieving pathological complete response (pCR) based on the FAO signature expression for each neoadjuvant chemotherapy trial indicated

As shown in Fig. 3a, pre-treatment tumour samples from 102 patients [24] who received short term oestrogen deprivation treatment and achieved complete response as determined by the Response Evaluation Criteria In Solid Tumours (RECIST) had higher expression of the FAO signature, compared to patients who progressed (Wilcox rank sum test *p* = 0.002). We also determined the odds ratio between the FAO signature expression and pathologic complete response (pCR) to chemotherapy using published gene expression data from six neoadjuvant chemotherapy trials [25–30]. As summarised in Fig. 3b, patients with low FAO signature expression had greater odds of achieving pCR, compared to patients in the high group (average odds ratio 2.94, 95% confidence interval 1.38–6.82). Taken together, these data suggest that the FAO signature expression in primary breast tumours is associated with response to both neoadjuvant chemotherapy and aromatase inhibitor therapy.

*Expression of FAO signature is prognostic in other cancer types* – To investigate whether the prognostic performance of the FAO signature could be extended to other tumour types, we explored whether the signature expression was associated with prognosis in an additional six different cancer types.

**Fig. 4:**
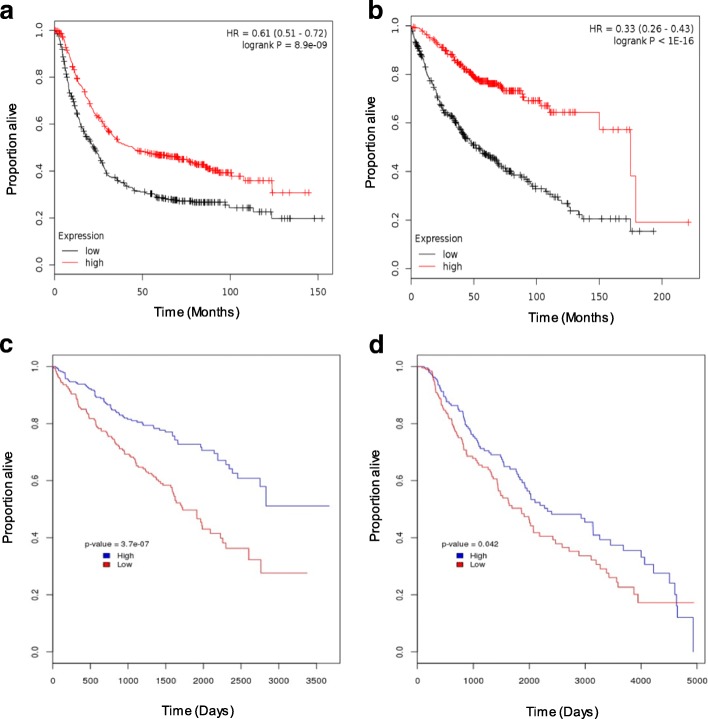
FAO signature expression is prognostic in different cancers. High expression of the FAO signature in **a** gastric (*n* = 876, log-rank *p =* 8.9E-09) and **b** lung adenocarcinoma (*n* = 720, log-rank *p <* 1.1E-16) is associated with good overall survival in the KMplotter cohort. High expression of the FAO signature in the TCGA **c** ccRCC (*n* = 588, log-rank *p =* 3.7E-07) and **d** melanoma (*n* = 396, log-rank *p* = 0.042) cohorts is correlated with good overall survival. High and low expression was defined as patients with average FAO signature expression value above and below the median cutoff, respectively

The FAO signature was highly prognostic in gastric (Fig. 4a, log-rank *p* = 8.9e-09) and lung adenocarcinoma (Fig. 4b, log-rank *p* < 1.1e-16). We also analysed clear cell renal cell carcinoma (ccRCC) and melanoma cohorts from TCGA and observed high FAO signature expression to be associated with better prognosis (Fig. 4c, ccRCC log-rank *p* = 3.7e-07; Fig. 4d, melanoma log-rank *p* = 0.042). However, in colorectal and ovarian cancers, no significant differences in survival were observed between the FAO signature expression and outcome (Additional file 2: Table S2, Additional file 3: Fig. S1). These data suggest that the FAO signature expression is prognostic in several other different cancer types, in addition to breast cancer.

**Fig. 5:**
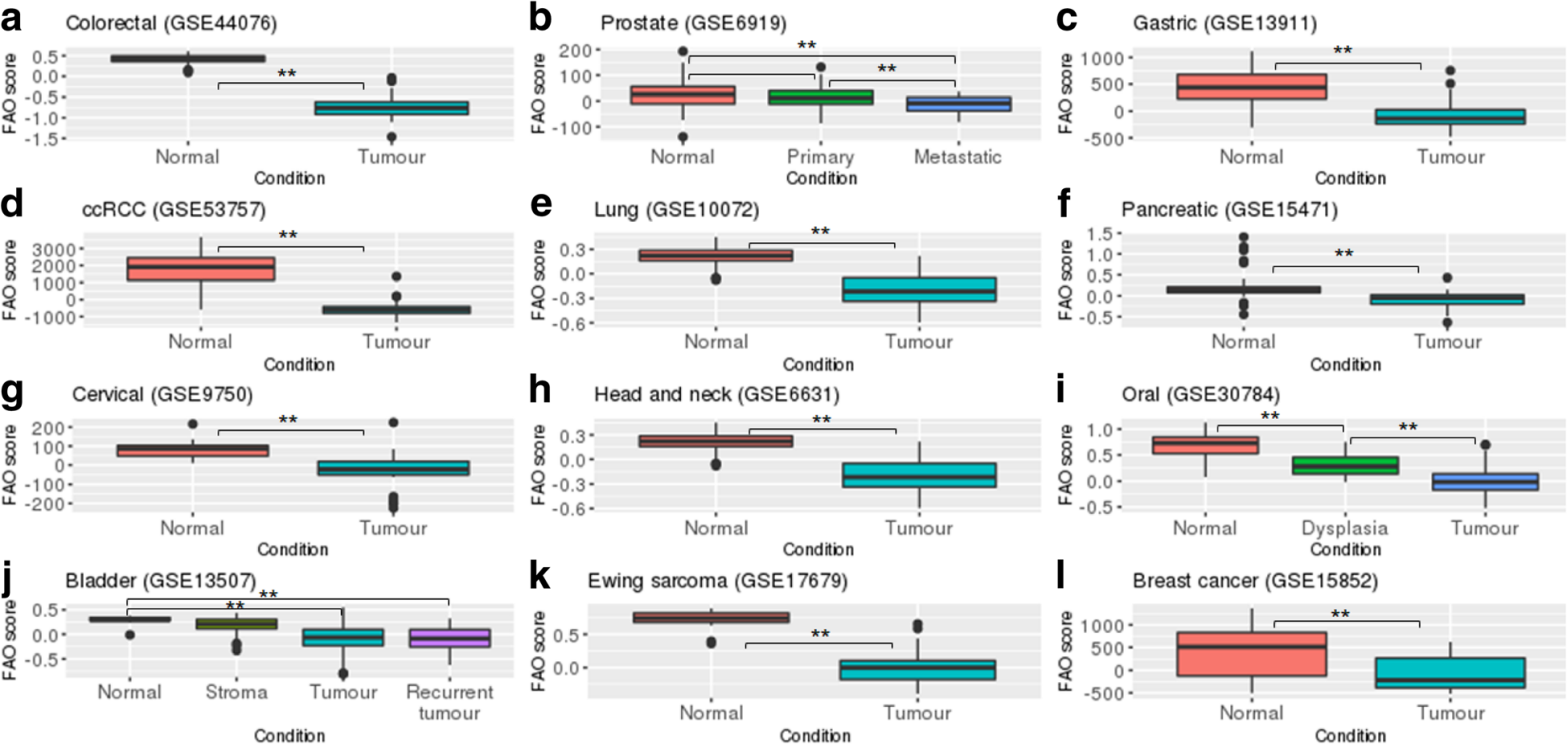
FAO signature is expressed at higher levels in normal compared to tumour tissues from different sites. Gene expression datasets from tumour and normal tissues were accessed and analysed for expression of the FAO signature. ** Wilcoxon rank sum test *p* < 0.01; NS, non-significant

*FAO signature expression is lower in tumour, compared to non-cancerous tissues* – The observed association between the FAO signature and prognosis in some cancer types raises the question as to whether expression of this signature is altered in tumour, compared to non-tumour or normal tissues. To address this question, we analysed the FAO signature expression across tumour and non-tumour tissues using publicly available gene expression datasets from multiple cancer types. As shown in Fig. 5, the FAO signature expression was consistently downregulated in tumours, compared to non-tumour tissues in all datasets analysed. In prostate cancer, we observed a trend of decreased expression of the FAO signature between non-tumour and primary tumour tissues, which achieved statistical significance when the former was compared to metastatic tissues (Fig. 5b). In oral cancer, the FAO signature expression was lower in benign dysplasia and primary tumours, compared to non-tumour tissues (Fig. 5h). These data suggest that tumours from various anatomic sites downregulate the expression of the FAO signature compared to normal, healthy tissues.

*Expression of CPT1A is expressed higher in ER-positive, compared to ER-negative breast tumours and cell lines* – Based on our computational findings, we sought to understand how modulating FAO affects breast cancer cell biology. Since CPT1A is a member of the 19-gene signature and the rate-limiting enzyme in FAO, we modulated the expression of this enzyme in vitro. Of note, genetic modulation and pharmacologic inhibition of CPT1A has been shown to alter FAO flux [31–33]. Since low FAO signature expression in breast tumours was associated with poor outcome, we investigated the effect of CPT1A overexpression in a breast cancer cell line.

**Fig. 6:**
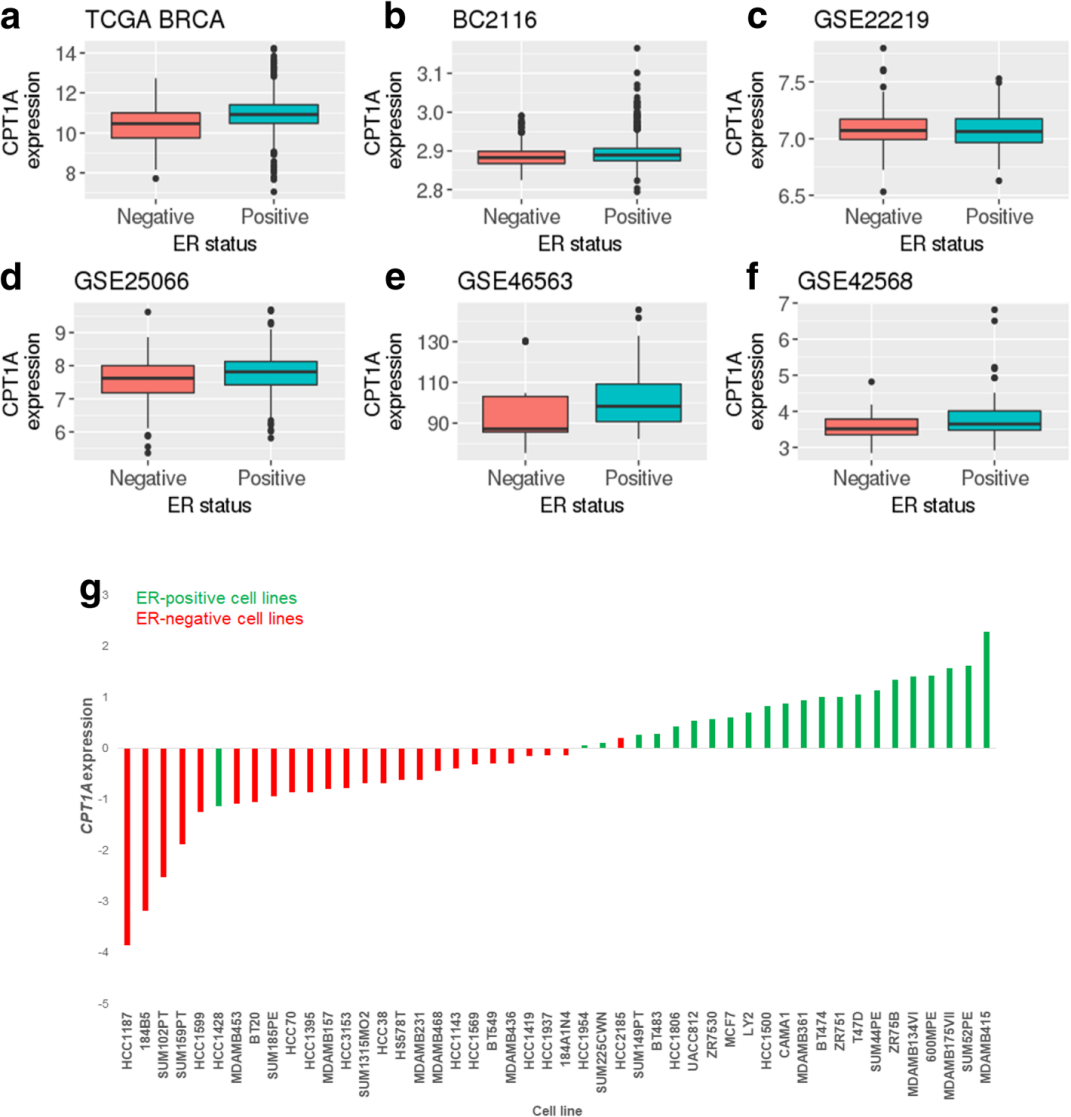
Expression of *CPT1A* is expressed at higher levels in ER-positive, compared to ER-negative breast tumours and cell lines. **a**-**f** *CPT1A* is expressed higher in ER-positive than ER-negative tumours in 5 of 6 datasets analysed. **g**-**h** In two independent datasets, ER-positive (green bars) breast cancer cell lines generally exhibit higher *CPT1A* expression, compared to ER-negative (red bars) cell lines. ER-positive vs ER-negative Wilcoxon rank sum test *p* < 0.01 for both datasets. ** Wilcoxon rank sum test *p* < 0.01

First, we surveyed the expression levels of *CPT1A* in breast cancer tissues and cell lines. Five out of six primary breast tumour gene expression datasets (details in Additional file 1: Table S1) analysed showed significantly lower *CPT1A* expression in ER-negative, compared to ER-positive tumours (Figs 6a-f). Consistent with the findings observed in tumour tissues, analysis of published microarray gene expression data from a panel of breast cancer cell lines revealed a striking enrichment of lower *CPT1A* expression in ER-negative, compared to ER-positive cell lines (Fig. 6g Wilcoxon rank sum test *p =* 5.41e-10; Fig. 6h Wilcoxon rank sum test *p* = 3.49e-09). This finding was also observed in an independent dataset, and additionally, *CPT1A* expression was decreased in an MCF7 cell line that is resistant to the chemotherapeutic agent adriamycin (Additional file 4: Fig. S2, black arrow), compared to the wildtype parental line. Based on data from the in silico analysis, the ER-negative MDA-MB231 cell line, which has low mRNA expression of *CPT1A*, was selected for overexpression analysis.

**Fig. 7:**
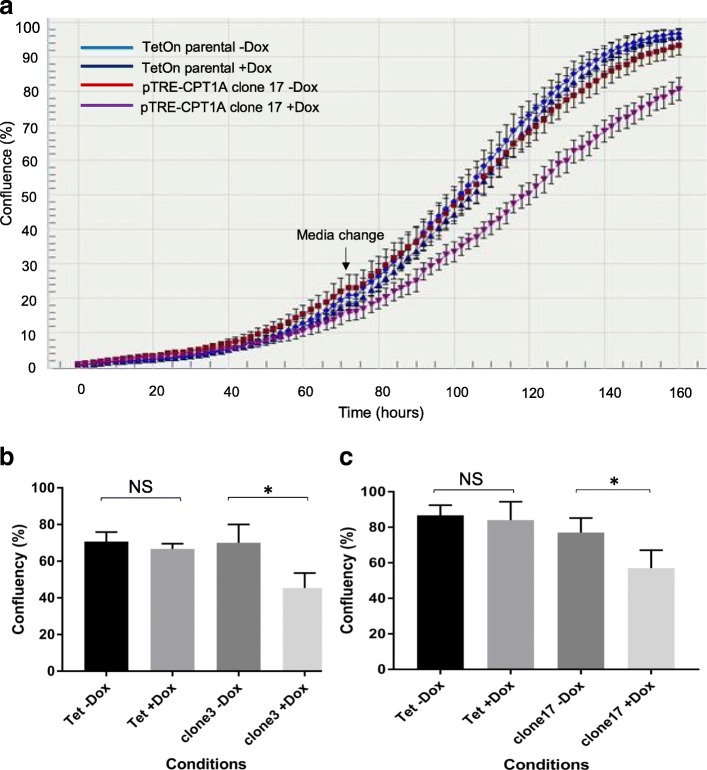
CPT1A overexpression decreases confluency of MDA-MB231 cell line. **a** Representative growth curve of MDA-MB231 pTRE-CPT1A cell lines with endogenous (blue circle) and CPT1A overexpression (navy blue triangle). Error bars = standard deviation. **b**, **c** In two independently generated MDA-MB231 pTRE-CPT1A **b** clone 3 and **c** clone 17, CPT1A overexpression decreased confluency at 120 h (n = 3, error bars = standard error of mean; * paired t-test -Dox vs + Dox *p* < 0.05; NS = non-significant)

*Overexpression of CPT1A decreases confluency in MDA-MB231 cell line* – As we had observed that the expression of the FAO signature is inversely correlated with proliferation in tumours, we investigated whether increased CPT1A expression altered the rate at which cultured cells achieved confluency. We generated a doxycycline-inducible system to overexpress CPT1A in the MDA-MB231 cell line [34, 35]. Characterisation of the induction of CPT1A by western blot analysis is this shown in Additional file 5: Fig. S3. Cells were pre-induced with doxycycline for 48 h, and real-time growth kinetics monitored for one week. MDA-MB231 cells overexpressing CPT1A had an approximately 20–25% lower confluency than controls (paired t-test *p* < 0.05) in two independently generated pTRE-CPT1A cell lines (Fig. 7). This effect was not attributable to Dox treatment per se, as the control TetOn parental line had similar growth rates in the presence or absence of Dox (Figs 7b and c).

**Fig. 8:**
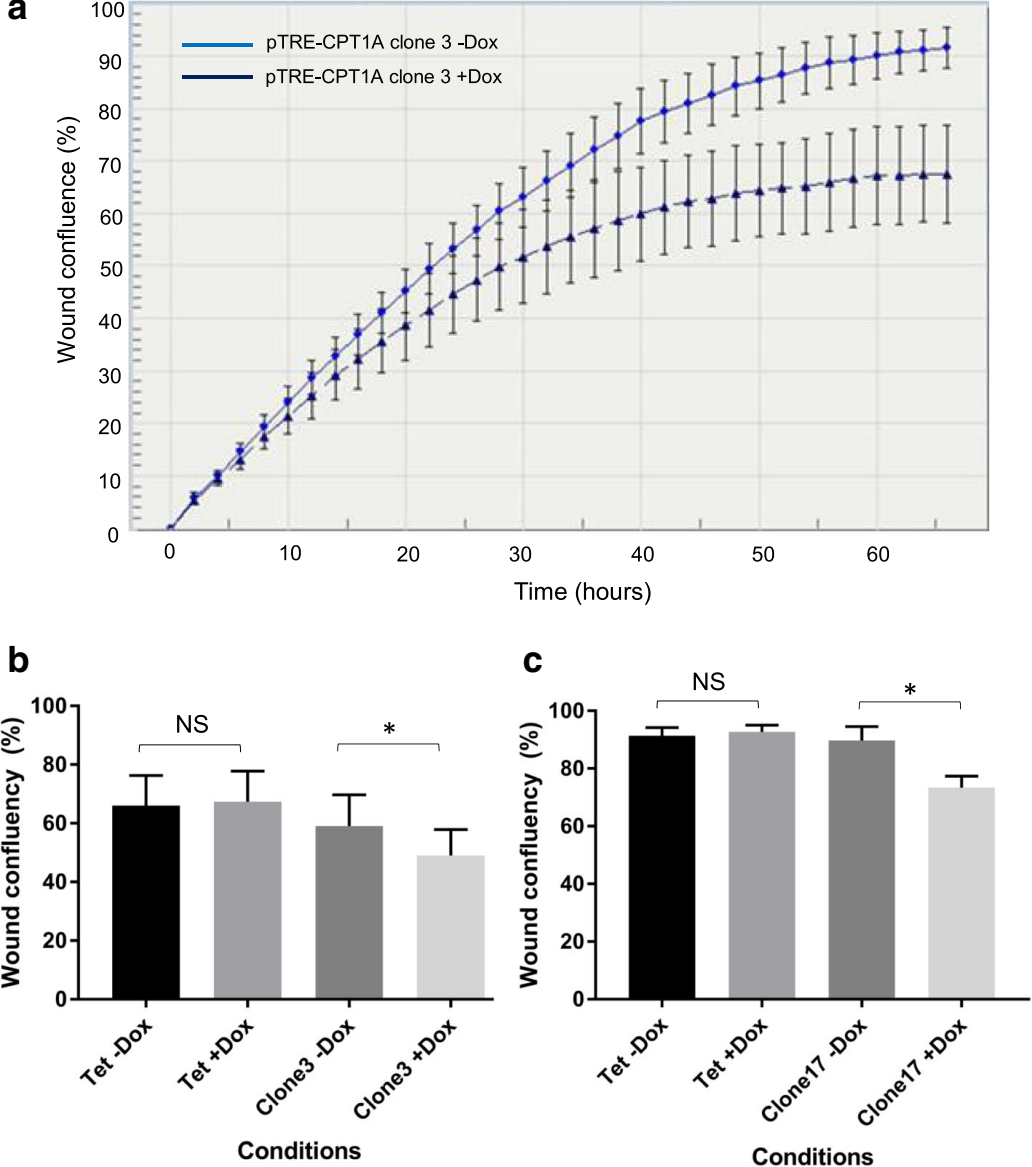
CPT1A overexpression decreases wound healing migration of MDA-MB231 cell line. **a** Representative wound closure curve of MDA-MB231 pTRE-CPT1A cell lines with endogenous (blue circle) and CPT1A overexpression (navy blue triangle). Error bars = standard deviation. **b**, **c** In two independently generated MDA-MB231 pTRE-CPT1A **b** clone 3 and **c** clone 17, CPT1A overexpression decreased wound closure rate (*n* = 3, error bars = standard error of mean; * paired t-test -Dox vs + Dox *p* < 0.05; NS = non-significant)

*Overexpression of CPT1A decreases wound healing rate in MDA-MB231 cell line –* We also investigated whether CPT1A overexpression altered gap closure rate in MDA-MB231 cells. Cells were induced for five days to express CPT1A and then seeded into 96-well plate at full confluency. A scratch was made through the middle of each well and gap closure rates between control and CPT1A overexpressing cells were monitored. As shown in Fig. 8, the wound closure rate was up to 16% slower in cells with CPT1A overexpression, compared to control (paired t-test *p <* 0.05). No significant difference in wound healing migration was observed between the Tet parental line in the presence or absence of Dox (Fig. 8b, c). Representative phase contrast images are shown in Additional file 6: Fig. S4.

**Fig. 9:**
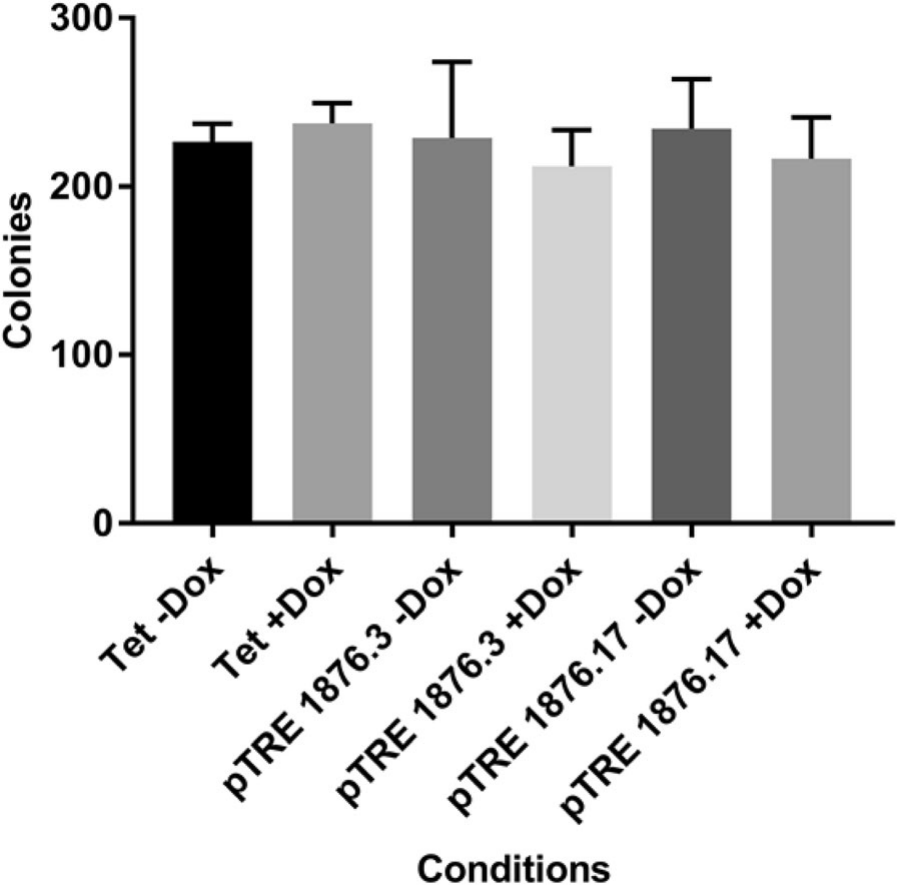
CPT1A overexpression does not alter anchorage-independent growth of MDA-MB231 cells. MDA-MB231 pTRE-CPT1A and TetOn parental lines were cultured in agar for two weeks and colonies counted under phase contrast. Number of colonies between -Dox and + Dox for TetOn parental and pTRE-CPT1A clones 3 and 17 were not statistically significant. n = 3, error bars = standard error of mean

*Overexpression of CPT1A does not influence clonogenic growth in MDA-MB231 cell line* – To investigate whether CPT1A overexpression in MDA-MB231 cells affects anchorage-independent clonogenic growth, we seeded and cultured cells in soft agar for two weeks, and counted colonies from each condition. As shown in Fig. 9, no significant difference in the number of colonies was observed between basal and CPT1A overexpressing MDA-MB231 cells.

## Discussion

This study sought to investigate genes and pathways that are associated with prognosis in breast cancer. Our bioinformatic analysis identified a gene expression signature composed of genes associated with FAO that was correlated with survival in some cancers. Cancer metabolism is an emerging hallmark of cancer [4]. Many studies have focused on understanding how glycolysis, glutamine metabolism and fatty acid synthesis affect cancer cell biology [7–9], however, the role of FAO in cancer remains contentious. Breast cancer cells treated with etomoxir to inhibit CPT1A resulted in cell death, while in vivo*,* mutant KRAS lung tumours were dependent on ACSL3-dependent FAO for tumour initiation and progression [36, 37]. However, findings from recent studies focused on human tumour samples and in vivo animal models suggest that activating FAO negatively affects tumour growth and progression [38, 39]. Here, we report a prognostic association between the expression of a gene signature involved in FAO and prognosis in some cancers. High expression of this signature was associated with better survival; and we validated this finding in multiple independent breast cancer datasets.

We observed that the FAO signature was expressed more highly in ER-positive and luminal molecular subtype breast tumours. This may be a result of the negative correlation between proliferation and the FAO signature expression, as ER-negative and basal/HER2-enriched molecular subtype tumours are generally more proliferative than ER-positive/luminal subtype tumours. However, Louie et al. previously demonstrated that the ER-negative MDA-MB231 cells incorporate exogenous palmitate into structural and signalling lipids, while the ER-positive MCF7 cells direct exogenous palmitate into acyl-carnitine – precursors of beta oxidation [40]. The different fates in response to exogenous palmitate may also explain why the FAO signature expression is lower in ER-negative, compared to ER-positive tumours.

In the neoadjuvant treatment setting, we found that ER-positive tumours from patients with high FAO signature expression had a better response to short-term oestrogen deprivation therapy. Additionally, patients with tumours that had low FAO signature expression that received pre-surgical chemotherapy had better odds of achieving complete response. The inverse correlation between the FAO signature expression and proliferation may explain why tumours with low expression of the FAO signature are more likely to achieve good chemotherapy response. In support of this finding, we analysed publicly available gene expression data from MCF7 cells with knockdown of ER expression, and observed higher expression of the MKS signature, and a trend towards decreased expression of the FAO signature, in cells with ER knockdown compared to control (Additional file 7: Fig. S5). Hence, these findings suggest a robust inverse relationship between cancer cell proliferation and the FAO signature expression.

We demonstrate in multiple tumour types that the FAO signature expression is downregulated in tumour, compared to normal, non-tumour tissues. In some tissue types, these findings are supported by the literature. In gastric cancer, Enjoji et al. performed a pilot analysis by qPCR and reported the expression of genes involved in FAO to be downregulated in tumours, compared to normal gastric tissues [41]. La Gory et al. reported that the expression of genes involved in FAO was lower in the 786-O ccRCC cell line, compared to normal kidney cells [42] and, recently, Du et al. reported that CPT1A expression is decreased in ccRCC versus normal kidney [43]. Here, we demonstrate that the FAO signature expression is lower in ccRCC compared to normal kidney tissues in multiple datasets. In colorectal cancer, proteomic profiling of normal colorectal tissue, benign adenoma and colorectal carcinoma found that the expression of enzymes involved in FAO were downregulated with advanced disease [44]. This finding supports our observation that the FAO signature expression is lower in colorectal carcinomas, compared to normal colorectal tissues. Notably, our data suggests a correlation between transcript abundance and the expression level of enzymes involved in FAO. Our finding is also consistent with the observation by Gaude et al. who reported a gene set involved in FAO to be downregulated in tumour, relative to normal tissues in 30% of cancer types examined [45]. Additionally, we observed the same trend in several other tumour types including lung, pancreatic, bladder and stomach adenocarcinomas, and oesophageal and renal cell carcinomas, which were not reported in the Gaude et al. study. Taken together, our analysis has shown the downregulation of the FAO signature expression in a broader range of tumour-normal tissues relative to that reported in the literature.

Expression of *CPT1A* – the gene encoding the enzyme catalysing the rate-limiting step in FAO – was lower in ER-negative, compared to ER-positive tumours in most of the datasets analysed. Furthermore, in silico analysis of gene expression data from a panel of breast cancer cell lines found that *CPT1A* expression was higher in ER-positive, compared to ER-negative cell lines. This is supported by the findings of Balaban et al., who reported the rate of oxidation of radiolabelled palmitate to be over 5-fold higher in MCF7 cells compared to the MDA-MB231 cell line [46]. It is important to note that in addition to major differences in ER expression between these two cell lines, they also have unique genetic aberration profiles [47]. Therefore, one can posit that changes in *CPT1A* expression may not just be driven by specific mutations or other genomic events, but rather, are part of the broader alterations that occur during tumour initiation and progression.

Overexpression of CPT1A in MDA-MB231 cells decreased their confluency by approximately 20–25%, and wound healing rate by 16%. These data suggest that modulating the rate-limiting enzyme of FAO can significantly alter the proliferation and migration rates of cancer cells. This decreased proliferation in MDA-MB231 cells in response to CPT1A overexpression is supported by two studies that modulate FAO by different means. Treatment of lung cancer cell lines with pioglitazone – an agonist of the nuclear receptor peroxisome proliferator activated receptor gamma – decreased the proliferation rates via increased FAO; which activated the tumour suppressor protein retinoblastoma to effect cell cycle arrest. In prostate tumours, Torrano et al. demonstrated that higher expression of *PPARGC1A* - a key transcriptional co-regulator that activates expression of genes involved in oxidative metabolism – was associated with favourable prognosis [39]. Importantly, overexpression of PGC1A (the protein encoded by *PPARGC1A*) increased FAO flux in PC3 prostate cancer cells, and decreased their proliferation and soft agar clonogenic growth. In another study, FAO flux analysis of RWPE-1 prostate epithelial cells and its increasingly invasive derivative lines found decreased FAO with increasing invasiveness [48]. Overexpression of CPT1A in MDA-MB231, however, did not affect clonogenic growth of the cells, compared to the control. One plausible explanation for this observation is that growth of cells under anchorage-independent conditions alter their metabolic requirements, which may differ from two-dimensional cell growth [49, 50]. Indeed, growth of the normal mammary epithelial MCF10A cells was impaired in part due to decreased glycolysis, and could be rescued by activation of FAO [49].

We acknowledge that our study is limited by the number of cell lines analysed for experimental characterisation of the bioinformatics findings. As such, future efforts could focus on modulating this pathway in cell lines from breast, as well as other cancer types, which could shed light on the common mechanisms as to how alterations in FAO affects cancer cell biology.

## Conclusions

In this study, we report an association between expression of genes involved in FAO and prognosis across multiple tumour types. Overexpression of CPT1A - the rate-limiting enzyme in FAO - decreased the proliferation rate of MDA-MB231 breast cancer cells. Hence, activation of this pathway in the adjuvant setting may improve treatment outcome, and future studies could explore the effect of modulating FAO in other cancers where the FAO signature has been shown to predict survival.

## Additional files

Additional file 1: **Table S1.** Published gene expression datasets analysed in this study. (XLSX 10 kb)

Additional file 2: **Table S2.** FAO signature expression and outcome in colorectal cancer datasets. (XLSX 10 kb)

Additional file 3: **Figure S1.** FAO signature expression is not associated with ovarian cancer overall survival from the KMplotter analysis. (PDF 180 kb)

Additional file 4: **Figure S2.** Expression of *CPT1A* lower in ER-negative, relative to ER-positive breast cancer cell lines. Black arrow indicates an adriamycin-resistant MCF7 cell line, which had lower *CPT1A* expression, compared to wild type MCF7 cells. (PDF 90 kb)

Additional file 5: **Figure S3.** Representative dose response and time course analysis of doxycycline induction of CPT1A expression in MDA-MB231 cells. (a) Dose response optimisation of doxycycline induction of CPT1A. Cells were seeded and induced with increasing concentrations of Dox from 0- to 5 μg/mL for 48 h. Twenty μg of proteins were resolved by SDS-PAGE, and immunoblotted for CPT1A expression. (b) Time course optimisation of Dox induction. pTRE-CPT1A clone 3 cells were seeded, induced with 2 μg/mL Dox for up to 96 h, and immunoblotted for CPT1A expression. (PDF 47 kb)

Additional file 6: **Figure S4.** Representative wound healing migration phase contrast images. The scratch wound is completely closed at 30 h in MDA-MB231 TetOn parental cells with Dox (a) or without (b) treatment. (c) The wound area in pTRE-CPT1A clone 17 -Dox clones were completely closed at 30 h, but not (d) cells induced with Dox. (PDF 117 kb)

Additional file 7: **Figure S5.** ER knockdown in MCF7 cells decrease FAO signature expression. MCF7 cells stably expressing shRNA against *ESR1* (gene encoding ER) had decreased expression of the (a) FAO signature, but increased expression of the (b) MKS proliferation signature. *n* = 3 for both basal and *ESR1* knockdown. ** t-test *p* < 0.01. (PDF 66 kb)

## Abbreviations

ACADM/SB/VL: Acyl-CoA dehydrogenase medium/short-branched/very long chain
ACSL3: Acyl-CoA synthetase long chain 3
ccRCC: Clear cell renal cell carcinoma
CoA: Coenzyme A
CPT1A: Carnitine palmitoyl transferase 1A
CPT2: Carnitine palmitoyl transferase 2
Dox: Doxycycline
ER: Oestrogen Receptor
FAIMoS: Functional Aromatase Inhibitor Molecular Study
FAO: Fatty acid oxidation
KEGG: Kyoto Encyclopedia of Genes and Genomes
METABRIC: Molecular Taxonomy of Breast Cancer International Consortium
MKS: Mitosis kinome score
pCR: Pathological complete response
PPARGC1A/PGC1A: Peroxisome proliferator-activated gamma coactivator 1A
qPCR: Quantitative polymerase chain reaction
RECIST: Response Evaluation Criteria in Solid Tumours
