# Supplementary figures and images for: Fatty acid oxidation is associated with proliferation and prognosis in breast and other cancers
Source: BMC Cancer. 2018 Aug 9;18:805. doi: 10.1186/s12885-018-4626-9 (PMC6085695; doi:10.1186/s12885-018-4626-9)

Supplementary Figure 1

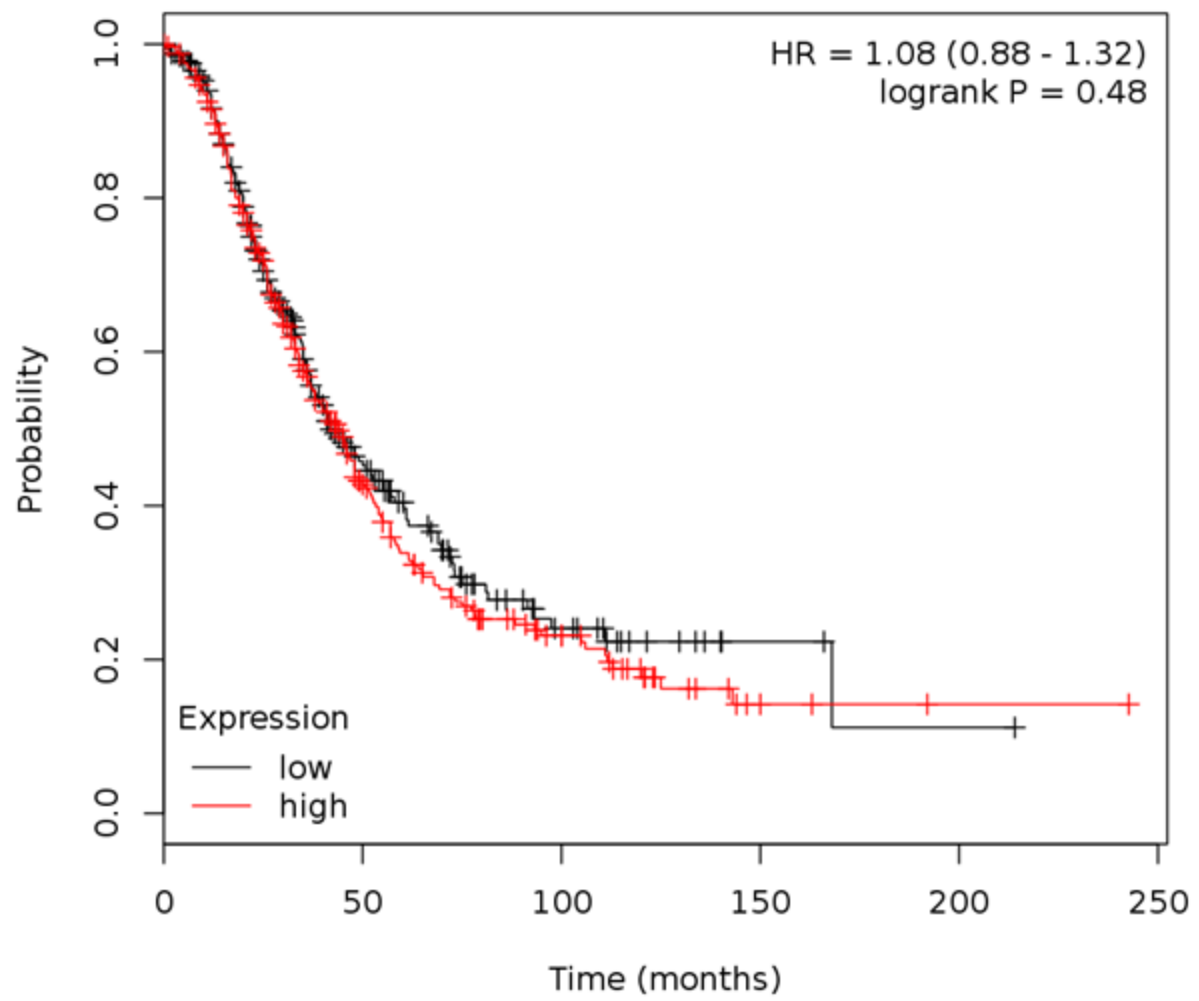

Supplement: Supplementary file 3 — Figure S1. FAO signature expression is not associated with ovarian cancer overall survival from the KMplotter analysis. (PDF 180 kb) [file 12885_2018_4626_MOESM3_ESM.pdf]

Supplementary Figure 2

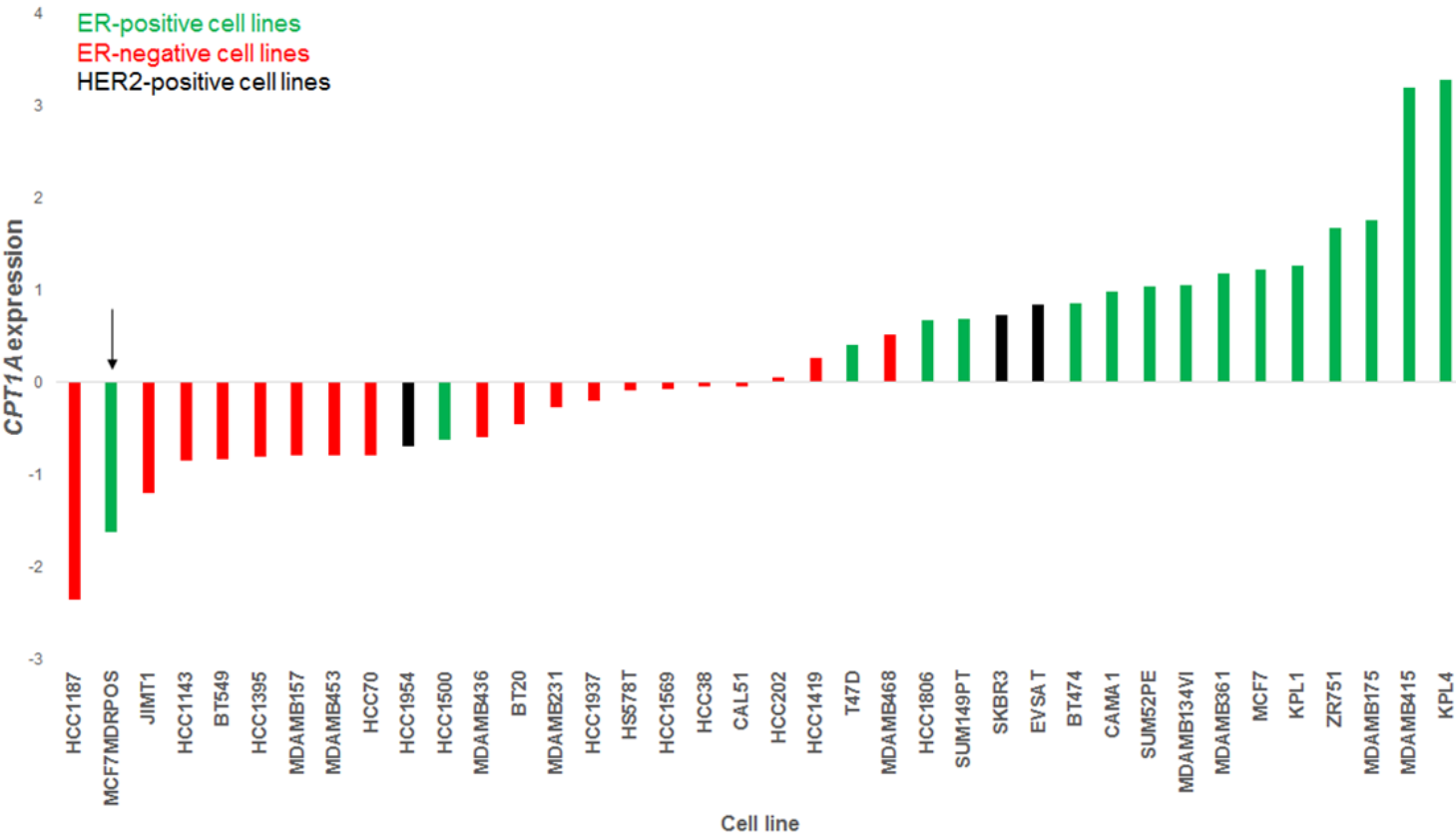

Supplement: Supplementary file 4 — Figure S2. Expression of CPT1A lower in ER-negative, relative to ER-positive breast cancer cell lines. Black arrow indicates an adriamycin-resistant MCF7 cell line, which had lower CPT1A expression, compared to wild type MCF7 cells. (PDF 90 kb) [file 12885_2018_4626_MOESM4_ESM.pdf]

Supplementary Figure 3

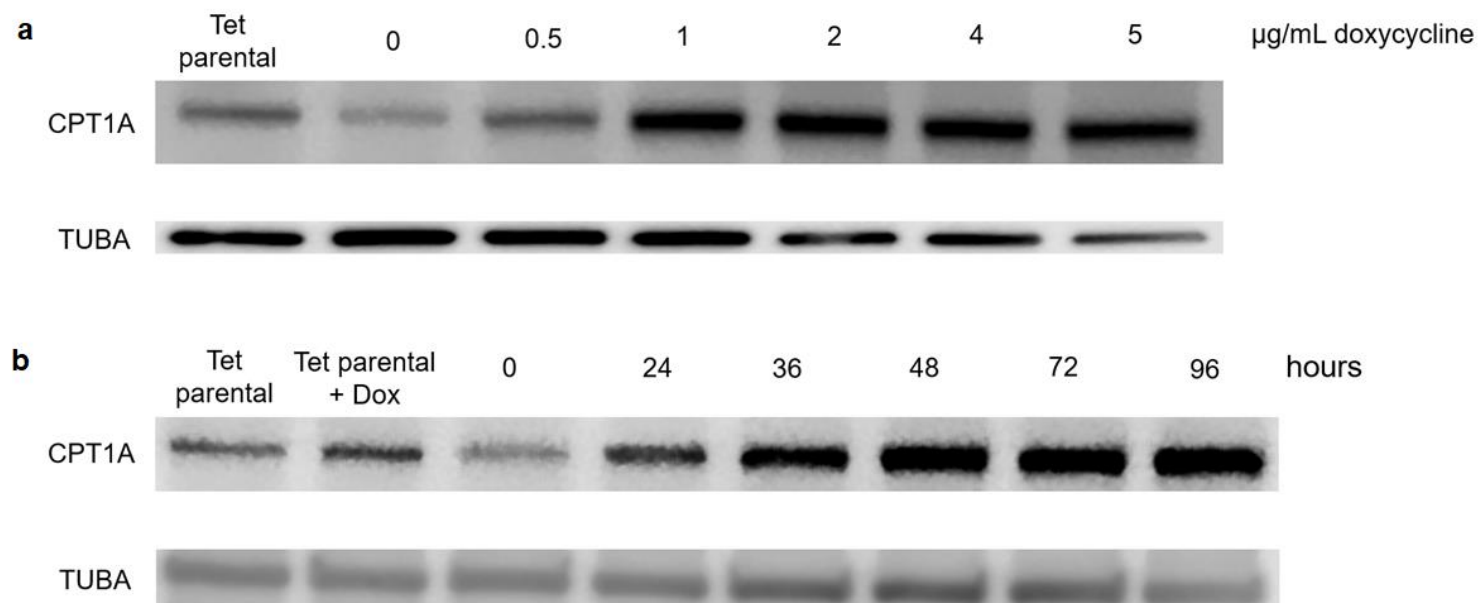

Supplement: Supplementary file 5 — Figure S3. Representative dose response and time course analysis of doxycycline induction of CPT1A expression in MDA-MB231 cells. (a) Dose response optimisation of doxycycline induction of CPT1A. Cells were seeded and induced with increasing concentrations of Dox from 0- to 5 μg/mL for 48 h. Twenty μg of proteins were resolved by SDS-PAGE, and immunoblotted for CPT1A expression. (b) Time course optimisation of Dox induction. pTRE-CPT1A clone 3 cells were seeded, induced with 2 μg/mL Dox for up to 96 h, and immunoblotted for CPT1A expression. (PDF 47 kb) [file 12885_2018_4626_MOESM5_ESM.pdf]

Supplementary Figure 4

**a**

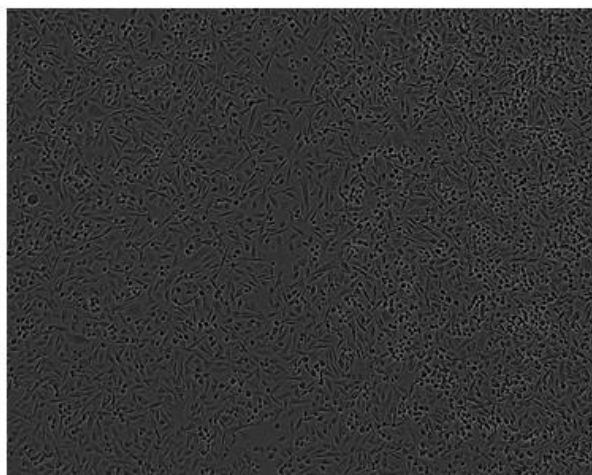

**b**

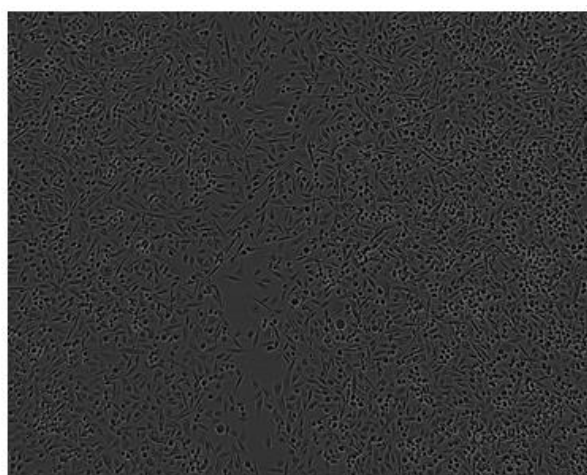

**c**

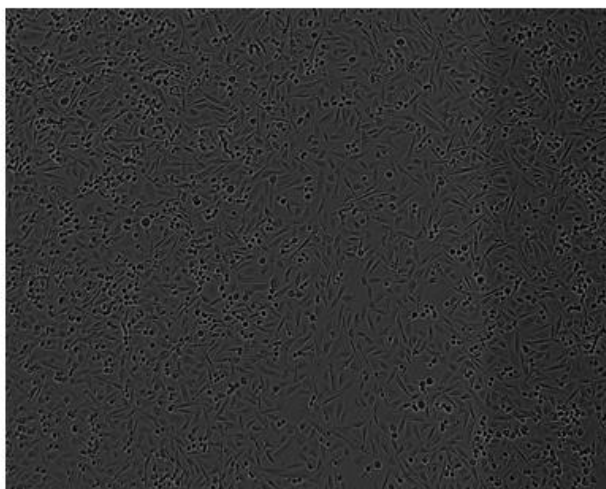

**d**

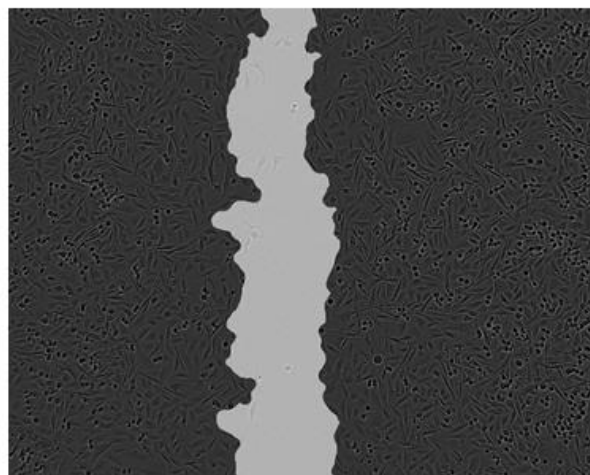

Supplement: Supplementary file 6 — Figure S4. Representative wound healing migration phase contrast images. The scratch wound is completely closed at 30 h in MDA-MB231 TetOn parental cells with Dox (a) or without (b) treatment. (c) The wound area in pTRE-CPT1A clone 17 -Dox clones were completely closed at 30 h, but not (d) cells induced with Dox. (PDF 117 kb) [file 12885_2018_4626_MOESM6_ESM.pdf]
