# Supplementary material for: Fatty acid oxidation is associated with proliferation and prognosis in breast and other cancers
Source: BMC Cancer. 2018 Aug 9;18:805. doi: 10.1186/s12885-018-4626-9 (PMC6085695; doi:10.1186/s12885-018-4626-9)

Supplementary Figure 5

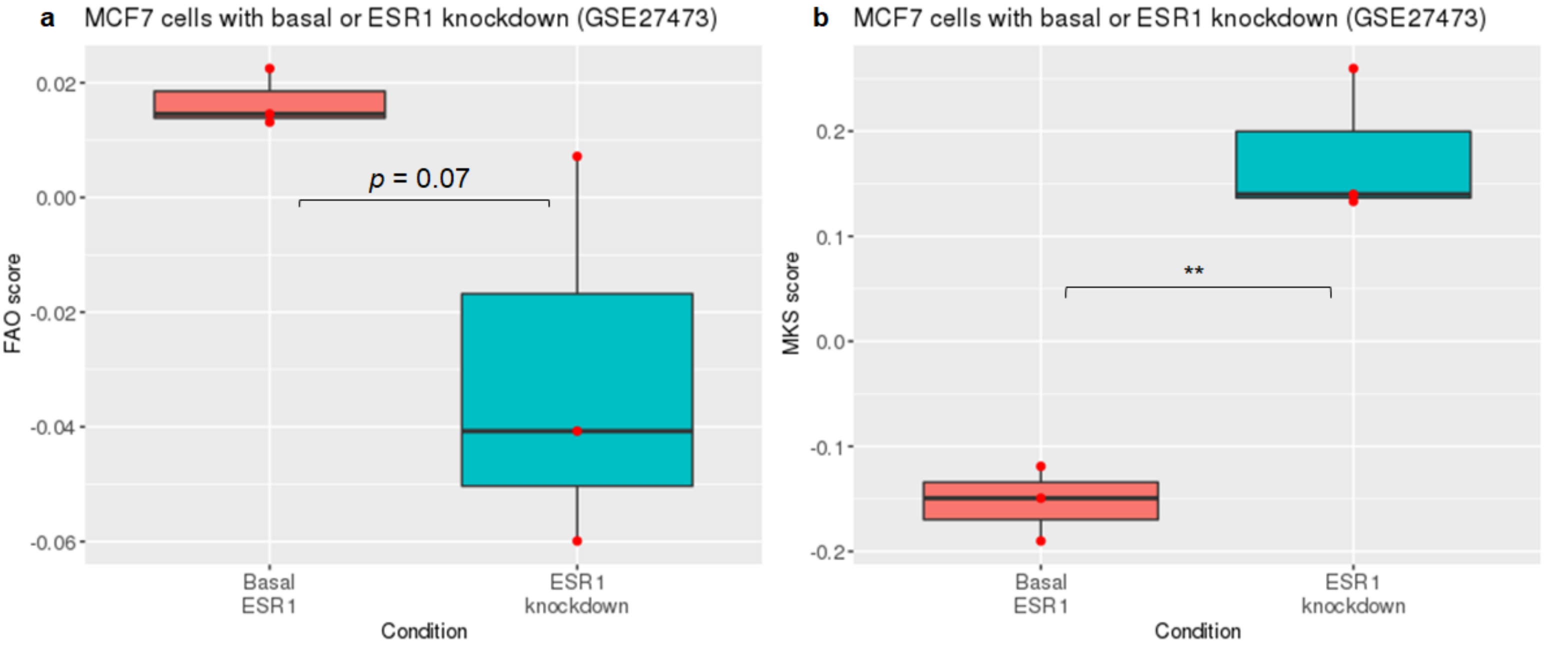

Supplement: Supplementary file 7 — Figure S5. ER knockdown in MCF7 cells decrease FAO signature expression. MCF7 cells stably expressing shRNA against ESR1 (gene encoding ER) had decreased expression of the (a) FAO signature, but increased expression of the (b) MKS proliferation signature. n = 3 for both basal and ESR1 knockdown. ** t-test p < 0.01. (PDF 66 kb) [file 12885_2018_4626_MOESM7_ESM.pdf]
